# Public Health Surveillance for *Australian bat lyssavirus* in Queensland**,** Australia, 2000–2001

**DOI:** 10.3201/eid0902.020264

**Published:** 2003-02

**Authors:** David Warrilow, Bruce Harrower, Ina L. Smith, Hume Field, Roscoe Taylor, G. Craig Walker, Greg A. Smith

**Affiliations:** * Queensland Health Scientific Services, Archerfield, Queensland, Australia; † Queensland Department of Primary Industries, Moorooka, Queensland, Australia; ‡ Public Health Services, Rockhampton, Queensland, Australia; § Queensland Parks and Wildlife Service, Rockhampton, Queensland, Australia

**Keywords:** Lyssavirus, Rhabdoviridae, public health surveillance, dispatch

## Abstract

From February 1, 2000, to December 4, 2001, a total of 119 bats (85 *Megachiroptera* and 34 *Microchiroptera)* were tested for *Australian bat* *lyssavirus* (ABLV) infection. Eight *Megachiroptera* were positive by immunofluorescence assay that used cross-reactive antibodies to rabies nucleocapsid protein. A case study of cross-species transmission of ABLV supports the conclusion that a bat reservoir exists for ABLV in which the virus circulates across *Megachiroptera* species within mixed communities.

Since the identification of *Australian bat lyssavirus* (ABLV) in bat species throughout Australia (*1*), public health units have been forced to confront its implications for human health. Fortunately, because of the close genetic and serologic relationship between rabies and ABLV, rabies immune sera and vaccines offer postexposure protection from infection (*1*,*2*). A bite or scratch from a bat in Australia constitutes a potential exposure to ABLV, and persons affected should be offered postexposure prophylaxis unless the bat can be shown to be uninfected with ABLV. However, such prophylaxis is costly and uncomfortable, and immune sera are in short supply worldwide. As many bats involved in such incidents are uninfected, and a negative results obviates the need for postexposure prophylaxis, determining each bat’s ABLV infection status is preferable. Under instruction from the public health units, Queensland Health Scientific Services Public Health Virology Laboratory has tested bats for ABLV since July 1998.

Two strains of ABLV are known to be circulating. One strain was isolated from a species of insectivorous *Microchiroptera*, *Saccolaimus flaviventris* (*1*). A second strain has been shown to infect the four species of *Megachiroptera* in the genus *Pteropus* that occur in mainland Australia (*1*,*3*). Isolates from the four pteropid species show minimal sequence variation with geographic origin and species and are essentially identical (I.L. Smith, unpub. data). Pteropid bats are nomadic nocturnal mammals that roost in trees during the day in colonies that frequently number in the thousands. Colonies may contain one or more species, and fluctuate in size, depending on available food resource (*4*). This dynamic social structure has been evoked to explain the circulation of a single strain of ABLV in pteropids (*1*).

In this article, we report on surveillance of bats brought in for testing to our laboratory. A case is described in which circumstantial evidence exists for bat-to-bat cross-species transmission of ABLV. This finding is consistent with a model in which the social structure of pteropid camps results in a single strain of circulating virus.

## The Study

Bats involved in incidents involving public health were tested for ABLV infection by immunofluorescence assay (IFA) on brain impression smears by using a fluorescein isothiocyanate-conjugated monoclonal (Fujirebio Diagnostics, Malvern, PA) or polyclonal (Biorad, Hercules, CA) antibodies to the nucleocapsid protein. The IFA was performed in parallel with a fluorescent real-time polymerase chain reaction (PCR) assay (*5*) for pteropid samples, or a heminested reverse transcriptase (RT)-PCR for all others (*6*). When it was possible, the bat was identified to the species level (*7*). For molecular analyses, the region encoding the carboxy-terminal of the glycoprotein and its long 3′ untranslated region were amplified by RT-PCR, and the products were directly sequenced by using Big-Dye chemistry (Applied Biosystems, Foster City, CA). Primers and reaction details are available on request.

During February 1, 2000, to December 4, 2001, a total of 119 bats, including 85 *Megachiroptera* and 34 *Microchiroptera,* were submitted for testing to the Public Health Virology Laboratory, Queensland Health Scientific Services. Bats submitted for testing had either bitten or scratched a person, or testing was considered to be in the interests of public health. Eight bats tested positive for ABLV infection by IFA (Table 1). Six of the bats positive for ABLV were *P. alecto* (75%); one bat positive for ABLV was *P. poliocephalus* (12.5%), and another bat that tested positive was an unidentified member of the genus *Pteropus* (12.5%). No positive *Microchiroptera* were obtained during the study period. Positive bats were from the Rockhampton area (37.5%), south of the Brisbane South metropolitan area (25%), Townsville area (12.5%), Sunshine Coast area (12.5%), and Brisbane South Coast area (12.5%). Confirmatory real-time or heminested RT-PCR results were concordant with IFA in all cases. Controls for contamination were negative for ABLV.

**Table 1:** Bats positive for *Australian bat lyssavirus* infection

Species	IFA positive^a^
*Megachiroptera*	
Pteropodidae	
*Pteropus alecto*	6/50
*Pteropus scapulatus*	0/18
*Pteropus poliocephalus*	1/8
*Pteropus conspicillatus*	0/4
Unidentified *Pteropus*	1/5
Subtotal	8/85
*Microchiroptera*	
Hipposideridae	
*Hipposiderosater*	0/1
Molossidae	
*Mormopterus beccarii*	0/2
*Mormopterus loriae*	0/2
Unidentified *Mormopterus*	0/2
Rhinolophidae	
*Rhinolophus philippinensis*	0/1
Vespertilionidae	
Chalinolobus gouldi*i*	0/1
*Miniopterus australis*	0/3
*Miniopterus schreibersii*	0/1
*Miniopterus scotorepens*	0/1
*Scotorepens orion*	0/1
Unidentified *Scotorepens*	0/1
Unidentified Vespertilionidae	0/14
Unidentified *Microchiroptera*	0/4
Subtotal	0/34
Total	8/119

^a^IFA, immunofluorescence assay; number positive/number tested.

In October 2000, a wild Black Flying Fox (bat 1, *P. alecto*) that was acting aggressively was removed from the top of a dome-shaped wire-mesh enclosure for viewing bats at a zoo in Rockhampton. Inside this enclosure were housed 23 flying foxes, all previously well. No new bats had been added to the cage for 12 months. The animal was euthanized and sent for testing to Queensland Health Scientific Services where it tested positive for ABLV by IFA on brain impression smear. One month later a captive Gray-headed Flying Fox (bat 2, *P. poliocephalus*) from within the enclosure was observed behaving abnormally. Normally a highly social animal, the bat was not moving freely and was licking its vulva profusely. The animal was relocated to an isolation cage where, during the next 20 hours, it exhibited a progressive neurologic syndrome. The bat was euthanized and found to be positive for ABLV by IFA.

To enable molecular epidemiologic studies to be carried out, the genomic RNA extracted from the brain of the two flying foxes mentioned previously, from another three other flying foxes from disparate locations (two Black Flying Foxes and one Little Red Flying Fox [*P. scapulatus*]), and from a person who acquired a fatal infection attributed to a flying fox were amplified across the variable noncoding intergenic region between the glycoprotein and polymerase. The reaction products were directly sequenced, and the differences are presented in Table 2. Of the six differences among the sequenced isolates, five were unique to both bats 1 and 2 submitted by the zoo. To date, ABLV sequence variation in flying foxes has been minimal across location, species, and time (I.L. Smith, unpub. data), so the identical variation seen in these two bats supports a model of natural cross-species bat-to-bat transmission.

**Table 2:** Nucleotide differences in the glycoprotein coding/noncoding region of *Australian bat* *lyssavirus* (ABLV)

	Nucleotide^a^					
Isolate host	4701	4751	4899	4987	5037	5058
*Pteropus alecto* ^b^	A	G	G	C	G	A
*Pteropus poliocephalus* ^c^	A	G	G	C	G	A
*Pteropus alecto* ^d^	C	A	A	T	A	G
*Pteropus alecto* ^d^	C	A	G	T	A	G
*Pteropus scapulatus* ^d^	C	A	G	T	A	G
Human^d^	C	A	G	T	A	G
*Pteropus alecto* ^e^	C	A	G	T	A	G

^a^Nucleotide position from the *Ballina* isolate of the pteropid strain of ABLV (8); A, deoxyadenosine; C, deoxcytidine; G, deoxyguanosine; T, thymidine.

^b^Case study, bat 1.

^c^Case study, bat 2.

^d^Queensland Health Scientific Services collection.

^e^From reference *8*.

The remaining captive bats in the enclosure were quarantined in a private facility off-site and were closely monitored for clinical, serologic, and behavioral changes during the next 3 months, and then for a further 3 months after they returned to their original enclosure. No attributable clinical disease, seroconversion, or behavioral change was observed during this time. The bat enclosure was modified to incorporate an extra outer mesh layer to prevent future direct contact with free-living wild bats outside the enclosure. Existing double-fencing had already prevented the public from having direct contact with flying foxes in the enclosure.

## Conclusion

The infection prevalence of 9.4% in submitted flying foxes in this study is not statistically significantly higher than that previously observed (6%) in sick, injured, and orphan flying fox submissions (3; H.E. Field, unpub.data); the wide 95% confidence interval (CI) (4% to 18%) reflects the limited sample size in this study. The prevalence is, however, statistically significantly higher than that observed in wild-caught flying foxes (3; H.E. Field, unpub. data), reinforcing the contention that the subpopulation of sick and injured flying foxes poses a higher risk for exposed humans. None of the submitted *Microchiroptera* was positive for ABLV. The small sample size in this study limits meaningful interpretation of this observation (the 95% CI for 0% prevalence with a sample size of 34 is 0% to 10%). Although this finding could indicate a lower incidence of ABLV in communities of *Microchiroptera*, downplaying the risk for human exposure posed by *Microchiroptera* in Australia would be premature.

The incident showing transmission from an ABLV-infected wild flying fox to a captive flying fox is interesting in two respects. First, an incubation period for the virus can be estimated. Assuming the scratch/bite occurred close to the time when the wild bat was discovered, the captive bat was observed to display symptoms after 29 days. This period compares with the only reported incubation time for an ABLV infection in a Black Flying Fox (*P. alecto*) of 6–9 weeks (*9*). Incubation times after experimental infection of Vampire Bats with rabies were shorter at 7–26 days for intramuscular injection (*10*) or 2–4 weeks after subcutaneous or intramuscular injection (*11*).

Second, this report is the first to describe probable natural cross-species transmission. This finding has implications for our understanding of how the virus circulates in bat communities. Evidence of cross-species transmission is consistent with the minimal sequence variation in isolates from sick or injured flying foxes obtained from various species at different sites around Australia (*1*). The large seasonally nomadic, multispecies colonies in which flying foxes commonly congregate (and interact) provide opportunity for interspecies and interregion transmission of ABLV. Our findings support this model for circulation of ABLV in pteropid colonies.
